# Bacteria and Tetanus

**Published:** 1887-08

**Authors:** 


### BACTERIA AND TETANUS.

In the November number of this journal, for 1882, a paper by Dr. Lester Curtis appeared, describing micro-organisms found in the blood of a tetanus patient. Up to that time no mention seems to have been made of any such organism in connection with this disease.

No direct conclusions were drawn from the observations then reported, but the impression conveyed was not favorable to the causation of the disease by the bacteria described, since they did not diminish in number with the improvement of the symptoms, and continued to exist in the blood after recovery was complete. They were shown in blood, freshly drawn from the finger, at a meeting of the Illinois State Microscopical Society, some weeks after the patient had resumed work and was apparently perfectly well. Since that time, in Europe, considerable attention has been drawn to the connection of micro-organisms and tetanus, but the statements of different observers by no means agree. A recent article by Doctor Bellmer, extending through two numbers of the *Berliner Klinische Wochenschrift*, for July 25th and August 1st, has recalled attention to the subject. The writer reports two cases of tetanus resulting in death; one of a man from a splinter of wood under the finger nail—the other, of a boy who drove a small piece of a sharp stone into the foot while going barefoot. In each of these cases inoculation-experiments were made with mice and guinea pigs. Splinters from the wood were trust into the sub-cutaneous tissue.

In the case of the boy, bits of the tissue from the neighborhood of the wound were inserted under the skin, and also samples of the earth from the play-ground where he received his injury. In every case tetanus followed. There seems, from these experiments, to be some evidence of toxic influence of the wood and the earth, although no mention is made of any control-experiments to remove doubt as to the possibility of other sources of the poison, as, for instance, impurities floating in the air.

Two sorts of bacteria are mentioned as having been found in the tissues of the patients: one a bacillus, and the other a micrococcus, both of which, so far as we can gather from the description, which is not very minute, and is without illustration, were described in Doctor Curtis' paper. The proof of the connection of these bacteria with the disease is promised but not given.

In this connection it may be remarked that Doctor Brieger (*Berliner Klinische Wochenschrift*, April 25th, 1887) claims to have shown that several compounds belonging to the ptomaines produce tetanic symptoms, but that one in particular, which he names—"tetanin"—is the cause of the symptoms of tetanus. This substance seems to have been produced by the growth of bacteria. In this indirect way, bacteria may be the cause of the disease, though in themselves and apart from their product they may be harmless.

